# A Qualitative Study of Prescription Contraception Use: The Perspectives of Users, General Practitioners and Pharmacists

**DOI:** 10.1371/journal.pone.0144074

**Published:** 2015-12-03

**Authors:** Leigh-Ann Sweeney, Gerard J. Molloy, Molly Byrne, Andrew W. Murphy, Karen Morgan, Carmel M. Hughes, Roger Ingham

**Affiliations:** 1 School of Psychology, National University of Ireland, Galway, Republic of Ireland; 2 Whitaker Institute for Innovation and Societal Change, National University of Ireland, Galway, Republic of Ireland; 3 Discipline of General Practice, School of Medicine, National University of Ireland, Galway, Republic of Ireland; 4 Department of Psychology, Royal College of Surgeons in Ireland, Dublin, Republic of Ireland; 5 Perdana University Royal College of Surgeons in Ireland School of Medicine, Kuala Lumpur, Malaysia; 6 School of Pharmacy, Queen’s University Belfast, Belfast, Northern Ireland, United Kingdom; 7 Centre for Sexual Health Research, University of Southampton, Southampton, United Kingdom; University of St Andrews, UNITED KINGDOM

## Abstract

**Background:**

The oral contraceptive pill (OCP) remains the most popular form of prescription contraception in many countries, despite adherence difficulties for many. Uptake of long acting reversible contraceptives (LARCs), which are less reliant on user adherence, remains low. The aim of this study was to explore the experiences of, and attitudes towards, prescription contraception amongst samples of contraception users, general practitioners (GPs) and pharmacists.

**Methodology and Findings:**

We conducted a qualitative study using semi-structured interviews with 18 contraception users, 18 GPs and 9 pharmacists. The study took place in Galway, Republic of Ireland between June and September 2014. Thematic analysis was used to analyse the data. Overall, contraception users were more familiar with the OCP, and all the women interviewed began their prescription contraception journey using this method. All participants identified episodes of poor adherence throughout the reproductive life course. The identified barriers for use of LARCs were lack of information, misconceptions, lack of access and high cost. In contrast, GPs believed that adherence to the OCP was good and stated they were more likely to prescribe the OCP than other methods, as they were most familiar with this option. Barriers to prescribing LARCSs were time, cost to practice, training and deskilling. Pharmacists also believed that adherence to the OCP was generally good and that their role was limited to dispensing medication and providing information when asked.

**Discussion and Conclusion:**

There are contrasting perspectives between contraception service providers and contraceptive users. Training for healthcare providers is required to support informed contraceptive choice and adherence. It is necessary to address the practice barriers of cost and lack of time, to promote better communication around adherence issues and prescription contraception options. There is a need for more easily-accessible public health information to promote awareness on all methods of prescription contraception.

## Introduction

Unintended pregnancy has long been acknowledged as an important global health, social and economic problem which creates hardship for women and often impacts on the health and well-being of children. [1,2] There are considerable variations in the provision and receipt of prescription contraception services internationally. [3] For example, prescription contraception only became legal in Republic of Ireland (RoI) in 1979 under the Family Planning Act, which made contraception available on prescription through general practitioners (GPs) and pharmacists. [4] However, since the passage of this Act, contraception is now widely accessible in Ireland, and includes access to `emergency contraception`from pharmacies without a prescription since 2011. [5] Despite this progress in increasing the availability and uptake of contraception, unintended pregnancies are still a common occurrence in Ireland [6] and similar contexts in Western Europe. [2] GPs and pharmacists continue to be the gatekeepers to accessing the most effective methods of contraception; therefore, it is critical that they have a full understanding of contraceptive options and can assess and advise on their relative effectiveness.

The oral contraceptive pill (OCP) remains the most prescribed form of contraception in more economically developed countries internationally [3], despite evidence that poor adherence occurs throughout the reproductive life course. [7] This poor adherence significantly reduces the effectiveness of this method of contraception. [8] Nevertheless, uptake of long acting reversible contraceptives (LARCs) remains relatively low in Ireland and in other similar international contexts. [3,6,9,10] Data from other countries suggest that it is likely that there may be barriers to the provision of LARCs at the healthcare professional level [11–13]; these may include lack of skills in administration, poor knowledge and limited financial incentives. In order fully to understand patterns of prescription contraception use it is necessary to collect data from both those who use and those who provide access to these methods. There are few studies that have simultaneously gathered data from contraceptive users, GPs and pharmacists, despite increasing recognition of the important role played by each of these stakeholders in understanding effective contraceptive services and use. [11,14]

The overall aim of the study was to explore the experiences of, and attitudes towards, prescription contraception amongst users, GPs and pharmacists. The main objective of the study was to explore and elucidate the range of contraceptive user, GP and pharmacist factors that are likely to determine prescription contraception use and adherence.

## Methods

### Participants

Women using prescription contraception were purposively recruited in Galway city, in the west of Ireland, to represent various stages of the reproductive life course and various socio-economic backgrounds. The sample consisted of young university students, young mothers, older women with a third level (bachelor degree) qualification and older women from areas of higher social deprivation. These four groups represented the early and later stage of the reproductive lifespan and socioeconomic disadvantage as indexed by the participant’s age and the presence or absence of third level education. The university students and women with a third level qualification were recruited through the student and staff university intranet using an invitation e-mail that described the study. Women from areas of higher social deprivation were contacted through community based programmes using a `passive snowballing`technique to facilitate recruitment. This involved asking contacts in the community based programmes to discuss the research with those who they thought may be suitable or interested in volunteering to be participants. These participants were then given the contact details of the researcher so that they could independently volunteer to participate. This avoided any pressure being put on participants to take part in the study. This was the only group that was recruited in this manner, to ensure that the research sample overall did not know each other to avoid the closed group phenomenon [15] and, finally, young mothers were recruited through a `Teen Parenting Programme`in the community. GPs were recruited in Galway city and environ, through snowball sampling which aimed to recruit a broad sample of GPs representing urban and rural practices, male and females and with a range of years in practice. Using a similar approach, a smaller number of pharmacists were recruited through The Irish Medical Directory of pharmacies listings in County Galway. Snowball purposive sampling was also used to recruit participants from a similarly wide range as the GPs. This technique was used to encourage participation among these healthcare provider groups, as they are often a difficult to recruit population who receive multiple offers to be involved in research studies. The study research team advised that this method has been used with success in the past in recruiting health care providers who often refuse participation in studies when the invitation comes from unknown sources.

### Procedure

An interview topic guide was developed for contraceptive users to explore the experiences of, and attitudes towards, prescription contraception. This topic guide was based on key themes emerging from initial pilot interviews with a convenience sample of younger contraceptive users. This is outlined in Table 1. Semi-structured interview questions based on the emerging themes from the contraceptive users`interviews were designed for GPs and pharmacists. All the women were given information on the research in advance of their participation and they were asked to disclose their current method of prescription contraception to determine eligibility. Interviews took place in various locations, such as on the university campus and The Teen Parent Programme, which is based within the social work department, University Hospital, Galway. This programme works specifically with young mothers under the age of 20 years with a strong focus on parenting support and returning to education. The interviews with the women from areas of higher social deprivation took place in their homes, as this was most convenient for them with respect to travel and childcare concerns. Interviews with GPs and pharmacists were carried out at GP practices and pharmacy sites throughout the region. The interview topic guides that were used for the three groups are provided in Table 1. Use of this guide was led by the interviewees as they discussed their experiences in response to semi-structured ‘trigger’ questions. All interviews were carried out by the first author (LS).

**Table 1:** Interview topic guide.

Contraceptive users	Personal use of contraception
	What type of contraception do you use?
	Why did you choose this particular method?
	Were there any other influences that affected your decision to use this method?
	General knowledge of contraception
	Are there issues for you around the daily regime of the pill?
	Would you consider any other method of contraception such as LARCs?
	Would you say you are informed on all methods of contraception?
	Experiences of healthcare providers (GPs and pharmacists in particular)
	Can you tell me about your experience with your GP/Pharmacist?
	Were other methods and options explored?
GPs	Knowledge of the service user
	Can you provide a general profile of the women who access contraception within your practice?
	Do you think there is a preference amongst women seeking contraception for OCPs or LARCs?
	From your experience, can you comment on adherence of the pill?
	General role of the GP in prescribing contraception
	What is your role in prescribing contraception?
	Do you think you have any influence of contraception choices of patients?
	Can you reflect on how your biases towards certain methods of contraception when prescribing?
	Barriers and access
	Can you comment on the barriers that may exist for women in this community in accessing contraception?
	Can you comment on the barriers that you as a practitioner may face in prescribing contraception?
Pharmacists	Knowledge of the service user
	Can you provide a general profile of women who access contraception from this pharmacy?
	From your experience can you comment on adherence of the OCP, the daily regime?
	The general role of the pharmacist in dispensing contraception
	What is your role in dispensing contraception?
	Do you think you have think you have any influence over service users and their choice of contraception?
	Can you reflect how you might have biases towards any particular methods of contraception?
	Barriers and access
	Can you comment on the possible barriers that you as a pharmacist may face in providing contraception to service users?
	Can you comment on barriers that may exist for women in this community accessing contraception?

### Data analysis

All interviews were audio recorded and transcribed verbatim and analysed using thematic analysis; which was facilitated through the use of NVivo software. [16] This involved familiarisation with the qualitative data through repeated reading and note taking of the transcribed data from the interviews, generating initial codes, searching for themes, reviewing themes and finally defining and naming themes. The results are structured in terms of the main themes which emerged from the interviews. [17]

### Ethical approval

Ethical approval was provided by the NUI Galway Research Ethics Committee (Reference number: 14/JAN/03). All participants provided written informed consent to participate in the study and this was approved by the ethics committee.

## Results

Forty-five face-to-face interviews were conducted in three stages with the three separate groups of participants, prescription contraceptive users, GPs and pharmacists. Sample data in the form of interview quotes are integrated into the findings in *italics* to illustrate the participants experiences [18].

### Contraception users

A stratified purposive sample of 18 prescription contraception users shared their experiences of past and current prescription contraception use. The participant profile is summarised in Table 2. The main themes that emerged from the analysis of these experiences were socio-economic status, knowledge and attitudes towards LARCs and relationships with healthcare professionals, in particular with GPs and pharmacists.

**Table 2:** Contraceptive users in the study (n = 18).

Participant profile	Number of women	Age range
University students	5	(18–23 years)
Working women who held a third level qualification	4	(26–46 years)
Young mothers	4	(18–20 years)
Women from areas of higher social deprivation	5	(26–40 years)

Socio-economic determinants of contraception use: Of the 18 women interviewed it became clear that there were similarities and differences depending on socio-economic circumstances. The young mothers and the women from areas of higher social deprivation were all using LARCs at the time of interview. All of these women disclosed an unplanned pregnancy in their late teens as a consequence of poor adherence to the OCP, either due to forgetting to take their OCP, and/or using the OCP incorrectly. The women stated that their pregnancies had not been planned and that they did not feel they were prepared emotionally or economically for parenting. At the time of their pregnancy, they stated that they were in insecure relationships or alone, and were dependant on informal (family and friends) or formal support (community support and healthcare professionals) to manage the parenting role.

Only after giving birth did the women learn of LARCs as a contraception option from their GP or during their anti-natal care. The young mothers had all decided to use LARCs following pregnancy. However, the older women—over 30 years of age—from areas of higher social deprivation disclosed further unintended pregnancies at a young age as they were unaware of LARCs as a contraception option until more recently and were using the OCP up until this point. Difficulties with adherence continued while trying to balance the commitment of remembering to take the oral contraceptive pill daily, while looking after a small baby.

*like I mean too much responsibility*, *I had to look after a child*, *yeah and like you said even adding in another pill or adding in something else*, *it was just too much*, *too much to think about`*(Participant from an area of higher social deprivation)

By using LARCs, the women talked about how they were no longer afraid of becoming pregnant which gave them a sense of `*looking to the future*`,

*You know like*, *but I haven’t had any* (pregnancies) *since I got it in* (Mirena^®^ coil) *you know… it has worked wonders for me and I wouldn’t change it for the world…* (Participant from an area of higher social deprivation)

The decision to use LARCs helped the women to view their environment differently. This group believed they were advocates for LARCs in their community, in particular, talking to young women at risk of unintended pregnancies in an informal capacity. The women wanted other women in their community to become informed on LARCs and to recognise the consequences of the OCP and poor adherence.

The university students and women holding a third level qualification were using the oral contraceptive pill. Similar to the women who were young mothers and women from areas of higher social deprivation, they all began their prescription contraception journey with the oral contraceptive pill. This group in particular, however, had not experienced an unplanned pregnancy and therefore had not thought about changing their contraception, and they continued to use the oral contraceptive pill despite disclosing poor adherence throughout the life course.

Knowledge and attitudes about contraceptive methods: For the women holding a third level qualification, their entry into contraception use was similar to that of the university students. However, as long term oral contraceptive pill users, they were reluctant to change to another prescription method.

*If I had to make a choice in the morning*, *I think I would come off contraception altogether as opposed to go on an alternative like the Merina…* (Participant Working and held a third level qualification)

The student women and the women who held third level qualifications were less informed about LARCs.

*Just*… *well me and my friends we*, *like*, *don’t know what they are*, *don’t know how many other things are out there*… *how good are they are compared to the pill or are they the same*. (Student participant)

These groups held misconceptions about LARCs, such as `*infertility*`or `*delayed conception*`. The women didn’t like the idea of `*something long-term in their bodies*`and `*felt more in control*`using the OCP. Conversations about `*horror stories*`or bad experiences of LARCs through their social networks or the media were central to their choices.

*Yeah*, *and she has ballooned*, *she has put on so much weight and stuff*. *So she put me off that*. *So I don’t know*, *if I’m answering the question*. (Student participant)

On the other hand, young mothers and the women from areas of higher social deprivation were well-informed on LARCs and had a positive attitude towards them.

*I never notice it’s there*. *I think it’s great*. *I’ve had no problems with it*. *No*, *just a bit of spotting in the beginning…*. *And that was it*. (Participant who was a young mother)

*Supportive relationships for contraceptive choice and use*: The findings indicated that little discussion occurred between prescription contraception users and their GP about LARCs. The participants often relied on their friends and peers for information. The women shared experiences and knowledge with each other and often `*what their friends were on`*would influence their contraception choice. The younger women felt uncomfortable discussing contraception choice with their GP or pharmacist, often saying to their GP, `*No*, *I don’t like the idea of that*`, or, `*I just tell him/her everything is fine`*. Young OCP users talked about the social stigma they felt as contraception users. Young OCP users were not comfortable with anyone outside of their immediate social network knowing that they were using the OCP which also involved hiding their OCP from parents and other adults. These feelings extended to healthcare practitioners as they stated they felt `*they would be judged*`, or `*felt embarrassed*`talking to GPs or pharmacists.

*Yeah but just I’m very shy about that and I’m afraid to talk about it… When I was like 16*, *17*, *I would just go in*, *get it and run*, *very shy*, *didn’t want to ask many questions*, *yeah*. (Student, participant)

The women often refrained from asking questions or entering into discussions which were initiated by their GP, particularly when they were younger, as an opportunity to discuss adherence or other contraception options.

The older women felt no such discomfort talking to their GPs about contraception. However, they felt it was a personal matter and only did so if they had any concerns or were asked specific questions relating directly to their health or medical indicators associated with long term OCP use, such as smoking in their 40s. Despite adherence being poor across the reproductive life course for many women, they were less likely to disclose this to their GP or pharmacist and often `*doubled up on pills*`or took their OCP medication `*at different times*`, and withheld this information from both their GP and pharmacist.

Cost of LARCs: Where participants had to pay for LARCs and the related consultation, this was identified as potential barrier. Currently LARCs can cost approximately €300 in the Republic of Ireland, when the cost of the device, administration and related consultation fees are taken into account. Therefore, for lower income participants who do not qualify for free health care the OCP may be the only financially feasible option.

*Well like ideally I would love to be able to like fork out like two hundred euro to have like the implant or something*, *but as a student like I can’t really afford that*, *so I suppose it’s just like the easiest form for me to take*. (Student participant)

### GPs

We recruited 18 GPs in total. A summary of GP characteristics is provided in Table 3. These were mostly experienced practitioners as 16 of the GPs had more than ten years’ practice experience, while two GPs held five or more years of practice experience. The main themes which emerged from the analyses of the GPs experiences were how they distributed information to contraceptive users, the financial implications to their practice of delivering LARC services, access to training and maintenance of skills, and adherence awareness and responsibility.

**Table 3:** GPs in the study (n = 18).

Participant profile	n
Gender: Male/Female	10/8
Setting: Urban/Rural	9/9
Provided all LARC service	7
Provided contraceptive implant services	13
Training in all LARCs methods	9

Distributing information to contraceptive users: The GPs provided an insight into prescription contraception supply patterns. It was the practice of all GPs to provide information on all types of contraception; however, the quantity and quality of information given to women was inconsistent across practices. Information was often delivered in leaflet or booklet form, with the assumption that patient health literacy [19] was good. GPs felt that contraception choice was a personal matter for the patient and they would prescribe according to the patient’s wishes as long as their medical history did not suggest any contra-indications. The oral contraceptive pill was often requested first by the patient as that was the most familiar method known. Therefore, the oral contraceptive pill was the most prescribed method of contraception identified by all GPs in the study.

*I think the majority still express a preference for the oral contraceptive*. *It is the euphemism for contraception*, *oh it’s definitely the pill would be the preference… Oh there’s no doubt about it*. *It’s still probably I’d say 1 in 4 of them*, *that’s all that would be looking for long term thing*, *really*, *in the grand scale of things*. *In my practice*, *most just want the pill…* (GP participant)

Barriers for uptake of LARCs (Financial disincentives and training and maintenance of skills): From a practice perspective, the GPs outlined the barriers to providing contraception choice to their female patients. Cost was an important consideration because, in the Republic of Ireland, LARCs receive limited funding by the Government under a reimbursement scheme to insert intrauterine devices and the sub-dermal contraceptive implant. For this reason it is often not cost-effective for a GP practice to provide a comprehensive LARC service as there are poor financial incentives for doing so.

*`You know*, *if you’re not going to get funded for something*, *there’s no major incentive for you to start doing it*, *do you know what I mean*. *Yeah*. *And it’s exactly like that for me*. *I’ve 10 minute consultations do you know what I mean*. *So if I’m not getting incentivised in payment for it*, *if there isn’t a huge demand*, *if they can get it elsewhere*, *it’s not going to happen`*(GP participant)

GPs working in the community for 10 years or more were not necessarily trained to carry out LARC insertion procedures. Therefore, GPs were not always in a position to provide a LARC service. GPs had little incentive to become trained because of a cost deficit to their practice.

The GPs who were in practice for less than 5 years were trained in LARCs, due to the availability of training opportunities as part of GP training schemes in recent years; however, they were becoming deskilled as they are not in an environment where LARCs are being provided on a regular basis. The GPs stated that in their professional view, they would need to be `*carrying out at least two LARC procedures a week to remain skilled`*, but this was not often the case. Where GPs felt deskilled they would refer the patient to another practice or to a dedicated Family Planning Clinic. These are community-based health services that specialise in reproductive and sexual health, particularly the provision of contraception services.

Adherence awareness and responsibility: Significant discrepancies between the contraceptive users and the GPs were identified in the analysis; in particular, GPs’ understanding of adherence to the oral contraceptive pill. Despite all women interviewed in the study claiming to have issues of poor adherence throughout the reproductive life course, GPs felt that, overall, adherence was good and refrained from asking directly about adherence. Rather, they would ask more general statements such as `*how is everything going for you*?*`*, their main focus centred on their concern for side effects such as clots, headaches and monitoring blood pressure. Direct discussion between GP and patient on contraceptive adherence was generally avoided by both GPs and contraceptive user.

### Pharmacists

Nine pharmacists were recruited. This included two male and seven females, with five working in urban practices and four in rural practices. The analysis of the pharmacy data identified restrictions which impacted on the role of the pharmacist in prescription contraception delivery. The themes which emerged to identify this were legislation and practice, infrastructure and adherence awareness and responsibility.

Legislation and practice: Pharmacists believed that they were not currently equipped to meet the requirements to prescribe and monitor blood pressure as part of a comprehensive contraception service. In their view, this would require additional infrastructure within pharmacies and legislation change.

*Like our legislative abilities are kind of restricted with*, *you know what we can… We're obviously not able to prescribe*. *At the moment*, *it's obviously just a dispensing service and an advice offering service* (Pharmacist participant)

Further restrictions identified by pharmacists were that they do not have access to patients’ individual medical records or family medical histories, so they continue to remain hesitant to intervene or give advice on prescription contraception choice.

*Yeah*, *I think that deciding on which contraception is suitable for the patient*, *is between the patient and their GP* (Pharmacist participant)

The pharmacist in local community practices often felt that they had knowledge of a patient’s social circumstances, and could intervene or advise on contraceptive methods accordingly. However, they felt it was not their role to advise or prioritise social indicators over biological risk factors and felt that the initial assessment by the GP took precedence.

Infrastructure: Often in rural or small communities, the pharmacist worked alone and the option to discuss information or answers patients`questions was compromised. Likewise, pharmacists in busy urban locations discussed similar challenges and, despite having more than one pharmacist available at a time, they reported being too busy.

*We are very busy; often it is difficult to take time to talk to individual women*. *If a woman requests to speak with the pharmacist*, *yes*, *otherwise we don’t always have time…* (Pharmacist participant)

Adherence awareness and responsibility: Similar to GPs, pharmacists relied on providing information to the patient through leaflets or booklets as a means of addressing any queries or confusion the patient may have had. Thus, a similar assumption was made by pharmacists that patients both read and understand the literature. Pharmacists felt that providing literature was the best approach and they also assumed that adherence to the OCP for most women was very good. The pharmacists did not report women coming to them with concerns of poor adherence and felt that usually women ‘*were happy just to collect their prescription and go`*.

## Discussion

Our analysis indicated that there are significant gaps between the perspectives of contraceptive users, GPs and pharmacists on prescription contraception. In particular, the experiences of young mothers and women from areas of higher social deprivation did not reflect autonomy over their reproductive health before experiencing an unintended pregnancy. The participants felt awkward and self-conscious discussing contraception with their GP or pharmacist and did not feel confident in asking questions. GPs and pharmacists often assume that adherence is good and that contraception users fully understand the implications of their chosen method. A key finding is that ineffective communication with women about their prescription contraception was often the norm and that methods to promote adherence to OCP were undeveloped. GPs and pharmacists may need to ask more specific questions, such as “how are you finding taking the pill every day?”, to identify whether adherence is an issue for OCP users. [20] Given the known extent of non-adherence [7,21] it may also be appropriate to assume some level of non-adherence to the OCP and to ask, even before a disclosure of non-adherence, “What do you do when you miss a pill?” This raises the issue and acknowledges that missing the OCP is often a typical experience for those using this method of contraception.

This study also illustrates that the oral contraceptive pill is generally the GP’s first choice for in general practice when women initiate the use of prescription contraception. GPs were more familiar with the OCP and often less experienced in LARC insertions. The GP data also revealed similar concerns about skill maintenance in relation to LARCs that has been reported elsewhere. [10] Health care providers have a vital role in determining contraceptive use yet, in this study, GPs felt that poor financial incentives to provide LARCs services were a key structural barrier. There is evidence from the UK that changing financial incentives for GPs can increase the prevalence of LARCs use. [13] In the context of the Republic of Ireland, there is evidence that LARCs are more commonly used by women who qualify for free health care i.e. General Medical Services (GMS). [22] Those with GMS do not have to pay for LARCs and the associated general practice consultations. Therefore, LARC uptake may be unlikely to change significantly where there are financial disincentives for both those receiving and providing LARCs services.

The findings in relation to contraceptive user knowledge and attitudes about contraceptive methods are consistent with what has been found in qualitative studies in other similar contexts. [23–25] In particular, the misconceptions about LARCs and fertility may be difficult to change. Although even with the more familiar OCP, there is evidence that concerns and misconceptions about safety are common [22], therefore prescription contraception more broadly continues to be plagued by myths, which may negatively influence contraceptive choices and patterns of contraception use.

This study`s findings on adherence difficulties with the OCP concurs with studies that have shown poor adherence throughout the reproductive life course. [21,26,27] Contraceptive users participating in this study reported using their positive personal experiences of LARCs to promote interest and address misconception amongst their peers. Similarly, the participants in the study who had experienced an unplanned pregnancy as a direct consequence of poor adherence to the OCP, suggested sharing their experiences of the hardships they encountered both during and after an unplanned pregnancy, to promote adherence to the OCP and uptake of LARCs. Current evidence for intervention in this area is limited and requires more innovative approaches. [26]

The study also identified some social factors which impacted on contraception choices and experiences for prescription contraceptive users. In particular, the findings indicated the importance of supportive relationships for contraception use and choice. Therefore community-led contraceptive advice and support is one strategy that may warrant further investigation in intervention studies that aim to increase the uptake and adherence to contraception. [26] Young OCP users were not comfortable with anyone outside of their immediate social network knowing that they were using the OCP, as this was viewed to be an implied disclosure of sexual activity. This sometimes resulted in hiding their OCP from parents and other adults. The social determinants of contraceptive use require understanding of health behaviours both in light of the women’s immediate social context and also against the backdrop of current socio-political structures which impact on women’s health and well-being. [28]

The study findings can be used to inform further research, policy and practice. It is evident from the findings that a more comprehensive model of practice which is service user-led and cognizant of the social determinants of contraception experiences and choices are required. Further theory informed analyses of this qualitative dataset, e.g. use of the theoretical domains framework [29,30], can identify specific targets for complex behavioural interventions targeting contraceptive users, general practitioners and/or pharmacists. For example, it is clear from the thematic analysis of the present data that current financial incentives for both contraceptive users and general practitioners may not be optimal to support contraceptive choice and adherence. Studies from the UK [13] and USA [31] show that changing financial incentives leads to different prescription contraception choices. Randomised controlled trials of financial incentives e.g. free or reduced costs for those who pay for prescription contraception in Ireland, would reveal whether costs are significantly influencing prescription contraception choices. Ultimately, services that are designed to meet women’s needs will enable women to have a more active role in voicing their needs and preferences for contraception.

There are a number of limitations to the study. The findings are from a small qualitative sample confined to one geographical location, therefore generalising from these findings is limited by the study sampling. However, this is the first study to consider the perspectives of three key stakeholders in the provision and receipt of contraceptive services and has given an insight to critical structures and processes that appear to influence the uptake of and adherence to prescription contraception. Although this study took place in the Republic of Ireland, it is evident from the literature that aspects of the results are transferrable to other societies which share similar health-service, political and socio-economic structures. The results point to a number of specific areas where interventions could improve the uptake of and adherence to prescription contraception. [26]

## Conclusion

The findings show that there are some overlapping, and some contrasting, perspectives between prescription contraceptive users, general practitioners and pharmacists. These perspectives can influence choice and experience of prescription contraception and related services. Training for healthcare providers is required to support informed contraceptive choice and adherence. Improving contraceptive services requires due consideration of the social determinants of reproductive and sexual health. It is also necessary to address the limitations of cost and time to practice, which impact on communication between contraceptive users, GPs and pharmacists in order to assess adherence and time for counselling prescription contraceptive options. There is a need for further public health awareness on all methods of prescription contraception, which women can easily access and understand.
